# Biliary Microbial Structure of Gallstone Patients With a History of Endoscopic Sphincterotomy Surgery

**DOI:** 10.3389/fcimb.2020.594778

**Published:** 2021-01-27

**Authors:** Hongzhang Shen, Juanjuan Zhu, Fuqiang Ye, Dongchao Xu, Liangliang Fang, Jianfeng Yang, Huijie Lv, Qifeng Lou, Hangbin Jin, Ming Ni, Xiaofeng Zhang

**Affiliations:** ^1^ Department of Gastroenterology, Affiliated Hangzhou First People’s Hospital, Zhejiang University School of Medicine, Hangzhou, China; ^2^ School of Life Science and Technology, China Pharmaceutical University, Nanjing, China; ^3^ Department of Disease Control and Prevention, Center for Disease Control and Prevention of Eastern Theater Command, Nanjing, China; ^4^ The First School of Clinical Medicine, Nanjing Medical University, Nanjing, China; ^5^ Beijing Institute of Radiation Medicine, Beijing, China

**Keywords:** choledocholithiasis, bile, biliary microbiota, endoscopic sphincterotomy, amplicon sequencing

## Abstract

The biliary microbiota is related to the pathogenesis of human bile duct stones. However, the extent to which a history of invasive endoscopic sphincterotomy (EST) affects the biliary bacterial community remains largely unknown. We collected bile samples from the common bile duct of 100 choledocholithiasis patients. We performed 16S rRNA sequencing to investigate and compare the biliary microbial community. The patients without antibiotic treatment (AT) were grouped into three clusters based on their biliary microbial compositions. The patients with a history of EST were significantly enriched in one cluster mainly consisting of gastrointestinal bacteria compared with the other two clusters consisting of oral and environmental bacteria. The β-diversities of patients with and without EST were also significantly different, whereas the α-diversities were comparable. The only significantly enriched bacterial genus associated with a history of EST was *Pyramidobacter*, while eight other genera were significantly decreased. For patients with AT, seven of these genera maintained their association with EST, including *Pyramidobacter*. However, after AT, the difference in β-diversities was diminished. EST induced a marked shift in the biliary microbial composition. A cluster of biliary bacteria was associated with a history of EST, and *Pyramidobacter* was specific to EST.

## Introduction

Gallstones are a common disease of the human bile duct system. Approximately 15% of the population in the United States has cholelithiasis (Everhart et al., 1999), with choledocholithiasis patients accounting for approximately 10 to 20% of cholelithiasis patients (Williams et al., 2008). Common bile duct stone (CBDS) or choledocholithiasis can be divided into primary and secondary choledocholithiasis. Primary stones usually form in the bile duct and are prevalent in Asian populations, while secondary stones are common in Western countries. It is generally believed that primary choledocholithiasis could be caused by bacterial infection and cholestasis (Williams et al., 2008).

Researchers have endeavored to elucidate the biliary microbiota and its potential function in gallstone pathogenesis (Swidsinski and Lee, 2001; Stewart et al., 2002; Begley et al., 2005; Stewart et al., 2006; Boursi et al., 2016; Kose et al., 2018; Wang et al., 2018). Conventional techniques such as bacterial culture, polymerase chain reaction (PCR) targeting specific bacteria, and transmission electron microscopy have long been used to identify biliary bacteria including *Escherichia*, *Pseudomonas*, *Klebsiella*, *Enterobacter*, *Enterococcus*, *Haemophilus*, *Veillonella*, *Citrobacter*, and *Acinetobacter* (Brook, 1989; Swidsinski and Lee, 2001; Stewart et al., 2002; Yu et al., 2012). By using next-generation sequencing (NGS) technology, researchers can further reveal the composition and functions of microbiota and provide new insights into biliary bacteria. Wu et al. first applied 16S rRNA sequencing to bile and gallstone samples of patients with cholesterol gallstones (Wu et al., 2013). We previously performed unbiased metagenomic sequencing of bile samples from 15 patients with choledocholithiasis and identified 13 novel biliary bacteria (Shen et al., 2015; Ye et al., 2016). Recently, Kose *et al.* investigated the microbiota of pigmented and cholesterol gallstones from two patients (Kose et al., 2018).

Despite substantial advancements in the understanding of the biliary microbiota in recent years, the effect of invasive operations of the biliary duct, such as endoscopic sphincterotomy (EST), on the microbial community remains largely unclear. Although endoscopic retrograde cholangiopancreatography (ERCP) with EST is generally considered safe and effective, its invasive nature has been reported to be related to multiple short-term and long-term complications, including pancreatitis, cholangitis, recurrent CBDS, and post-procedural sphincter of Oddi inflammatory changes (Freeman et al., 1996; Tanaka et al., 1998; Konstantakis et al., 2017; Li et al., 2018; Nzenza et al., 2018; Zhou et al., 2019). It is generally believed that the function of the sphincter of Oddi cannot be well preserved after EST (Yasuda et al., 2001). The sphincter of Oddi plays an important role in protecting the biliary system from enteric bacterial invasion (Schneider et al., 2014). EST could lead to the dysfunction or damage of the sphincter of Oddi, which may further cause frequent duodenal-biliary reflux (DBR), i.e., regurgitation of the duodenal contents and gut bacteria into the bile duct, subsequent biliary tract infection and further choledocholithiasis recurrence (Sugiyama et al., 2004; Mu et al., 2015; Zhang et al., 2015).

Recently, Liang and colleagues performed 16S rDNA sequencing of bile samples from 18 cholangiolithiasis patients with sphincter of Oddi laxity (SOL) and found that they had more severe bacterial infections than patients without SOL (Liang et al., 2016). However, the infections did not differ between the patients with primary SOL or surgery-induced SOL. Comparatively, the biliary microbiota of patients with an intact sphincter undergoes a marked change in microenvironment after EST surgery, and thus, the alteration in the biliary microbiota in patients with a history of EST may be distinct from that in primary SOL patients. In this study, we focused on the influence of a history of EST on the biliary bacterial community and found EST led to a marked alteration in the biliary microbial composition which might be related with gallstone recurrence.

## Materials and Methods

### Study Design and Sample Collection

From October 2016 to January 2017, 100 patients who had been diagnosed with primary choledocholithiasis by computed tomography and B-mode ultrasonography at Affiliated Hangzhou First People’s Hospital, Zhejiang University School of Medicine were included in this study. These patients had no occurrences of gallbladder gallstones, hepatolithiasis, pancreatitis, or hepatobiliary tumors, otherwise they would be excluded from the enrollment. Patients with a history of cholecystectomy or diagnosed with secondary choledocholithiasis were also excluded from our study. Clinical metadata, including age, gender, BMI, antibiotic use, blood test results, and culture results of bile samples were also collected (**Table 1**). These patients were divided into four groups according to EST history and antibiotic treatment (AT) before the collection of bile samples (**Table 1**). (1) G_EST_, patients with an EST history and no AT, *n* = 20; (2) G_N_, patients without EST or other bile duct operation history and no AT, *n* = 31; (3) G_AEST_, patients with AT and had an EST history, *n* = 15; and (4) G_AN_, patients with AT and had no EST history, *n* = 34. For patients with antibiotic use, antibiotic treatment was ongoing at the timepoint of ERCP. All patients provided written informed consent upon enrollment. The study conformed to the ethical guidelines of the 1975 Declaration of Helsinki and was approved by the Institutional Review Board of Affiliated Hangzhou First People’s Hospital, Zhejiang University School of Medicine.

**Table 1:** Clinical patient characteristics.

Characteristic	G_EST_ (*n* = 20)	G_N_ (*n* = 31)	G_AEST_ (*n* = 15)	G_AN_ (*n* = 34)	*P* value
Sex (Female/Male)	7/13	16/15	9/6	19/15	Chi-square *P* = 0.413
BMI (mean ± SD)	21.92 ± 3.13	22.83 ± 3.28	22.32 ± 2.51	23.17 ± 3.93	Kruskal-Wallis *P* = 0.660
Age (mean ± SD) (yr)	67.10 ± 11.97	59.13 ± 16.79	73.73 ± 13.98	72.65 ± 7.94	Wilcoxon *P* (G_EST_, G_N_) = 0.123, Wilcoxon *P* (G_AEST_, G_AN_) = 0.703
White blood cell (mean ± SD) (×10^9^/L)	7.05 ± 3.86	8.38 ± 6.69	7.67 ± 3.19	6.88 ± 2.71	Kruskal-Wallis *P* = 0.778
Neutrophil granulocyte ratio (mean ± SD) (%)	74.31 ± 14.59	70.14 ± 14.83	72.89 ± 14.06	70.57 ± 12.94	Kruskal-Wallis *P* = 0.713
Hemoglobin (mean ± SD) (g/L)	122.85 ± 19.75	128.71 ± 17.56	124.53 ± 18.09	121.41 ± 112.36	Kruskal-Wallis *P* = 0.179
Blood platelet (mean ± SD) (×10^9^/L)	155.55 ± 52.72	174.94 ± 69.55	159.13 ± 53.30	177.88 ± 66.47	Kruskal-Wallis *P* = 0.626
Antibiotic use	0	0	15	34	
Third-generation Cephalosporin	0	0	11	26	
Broad-spectrum Penicillin	0	0	3	7	
Carbapenems	0	0	1	1	
Bile cultures*					
Positive	9	3	10	10	
Negative	3	13	3	12	
NA	8	15	2	12	

*G_EST_, patients with an EST history and no AT; G_N_, patients without EST or other bile duct operation history and no AT; G_AEST_, patients with AT and had an EST history; G_AN_, patients with AT and had no EST history; Negative, negative culture result; NA, culture not performed.

The samples were collected sterilely as previously described (Shen et al., 2015; Ye et al., 2016). In brief, strictly sterile side-viewing endoscopes (TJF240/JF-260V; Olympus Optical, Tokyo, Japan) were used to collect bile samples (2–5 ml) from each patient. Sterile sphincterotome catheters (V-SYSTEM; KD-V411M-0725; Olympus Optical), which passed through the work channel of the endoscopes, were used to aspirate bile samples from the common bile duct into sterile sputum cups. All samples were stored at −80°C until further use.

### DNA Extraction

Total DNA was extracted from 200 μl of each bile sample by using the Invitrogen Purelink Genomic DNA Mini Kit (Life Technologies, Carlsbad, CA, USA) according to the manufacturer’s instructions. The DNA concentration and quality were tested by NanoDrop 2000 Spectrophotometer (Thermo Scientific, Waltham, MA, USA).

### 16S rRNA Amplicon Sequencing

The V3-V4 region of the 16S rRNA gene was amplified by using the following primers: forward primer: 5’- TCGTCGGCAGCGTCAGATGTGTATAAGAGACAGCCTACGGGNGGCWGCAG -3’ and reverse primer: 5’- GTCTCGTGGGCTCGGAGATGTGTATAAGAGACAGGACTACHVGGGTATCTAATCC -3’. The libraries were constructed according to the protocol (https://support.illumina.com/downloads/16s_metagenomic_sequencing_library_preparation.html) for MiSeq 16S Metagenomic Sequencing Library Preparation (Illumina, San Diego, CA, USA) with the Phanta Max Master Mix Kit (Vazyme, Jiangsu, China). As previously described (Ye et al., 2016), we used three tubes of sterile water as negative controls during the library construction process. No DNA products were detected in the negative controls when evaluated by the E-gel electrophoresis system (Life Technologies). The libraries were then sequenced on a MiSeq platform (Illumina) to generate 2 × 300-bp paired-end reads. Each sample was sequenced for one time.

### Bioinformatic Analyses of 16S Sequencing Data

Quality control of the raw sequencing reads in “fastq” format was conducted by using cutadapt (v1.11). First, reads without V3-V4 primers were excluded. The pair-end reads were then merged into single sequences by PANDAseq (v2.9) (Masella et al., 2012) with an overlap of no less than 10 bp. Low-quality reads (with an average quality <Q20, having ≥1 N bases, or with a length <300 bp or >480 bp) were also filtered. Vsearch (v2.4.1) (Rognes et al., 2016) was employed to remove chimeras and cluster the remaining reads into operational taxonomic units (OTUs) at the 97% similarity level. Ribosomal Database Project classifier (v2.11) (Cole et al., 2009) then assigned a taxonomic rank (from phylum to genus) to each OTU. The analyses of the α- and β-diversities were performed by using QIIME (v1.8.0) (Caporaso et al., 2010). As previously described (Ye et al., 2016), taxonomic abundances and diversity indices were calculated by randomly resampling the reads of each sample to a uniform number (19,300 in this study) 1,000 times to remove potential bias introduced by varying sequencing depth of these samples. The β-diversities were compared by using the script “compare_categories.py” in QIIME. The predicted profiles of Kyoto Encyclopedia of Genes and Genomes (KEGG) pathway and orthology terms were generated by Phylogenetic Investigation of Communities by Reconstruction of Unobserved States (PICRUSt) (Cox et al., 2014). A heatmap plot was drawn by using the pheatmap package in R with a base-10 logarithm transformation of each relative abundance (RA).

### Statistical Analysis

Categorical variables were tested by Chi-square or Fisher’s exact test in SPSS 22.0. Continuous variables were expressed as the mean ± SD when needed. Kruskal-Wallis and Wilcoxon rank-sum tests were employed to detect differences among samples from different groups in R (v3.5.1). When necessary, *P*-values were corrected to control the false-discovery rate (FDR) by using the method described previously (Benjamini and Hochberg, 1995). Differences with *P* < 0.05 or FDR < 0.05 were considered statistically significant.

## Results

### Patient Characteristics and 16S rRNA Sequencing

We included 100 patients with common bile duct stones and assigned them to four groups by history of EST and AT before sample collection. The distribution of gender, BMI, age, and the results of routine blood tests did not significantly differ between the relevant groups (**Table 1**). For patients without antibiotic use, 12 ones had positive bacterial culture results.

The bile samples of these patients were collected during ERCP and were submitted for 16S rRNA sequencing. We obtained 19,371 to 27,905 (mean 25,554) high-quality merged reads of the samples after quality control, chimera removal, and OTU clustering of the raw reads (**Table S1**). A total of 19,270 OTUs were identified and assigned taxonomic ranks in these samples. Rarefaction curves measured by the Chao1 and Shannon indices revealed that the sequencing depth reached a plateau as the read number increased (**Figure S1**).

### The Biliary Microbiota of Patients Without Antibiotic Treatment

**Figure 1:**
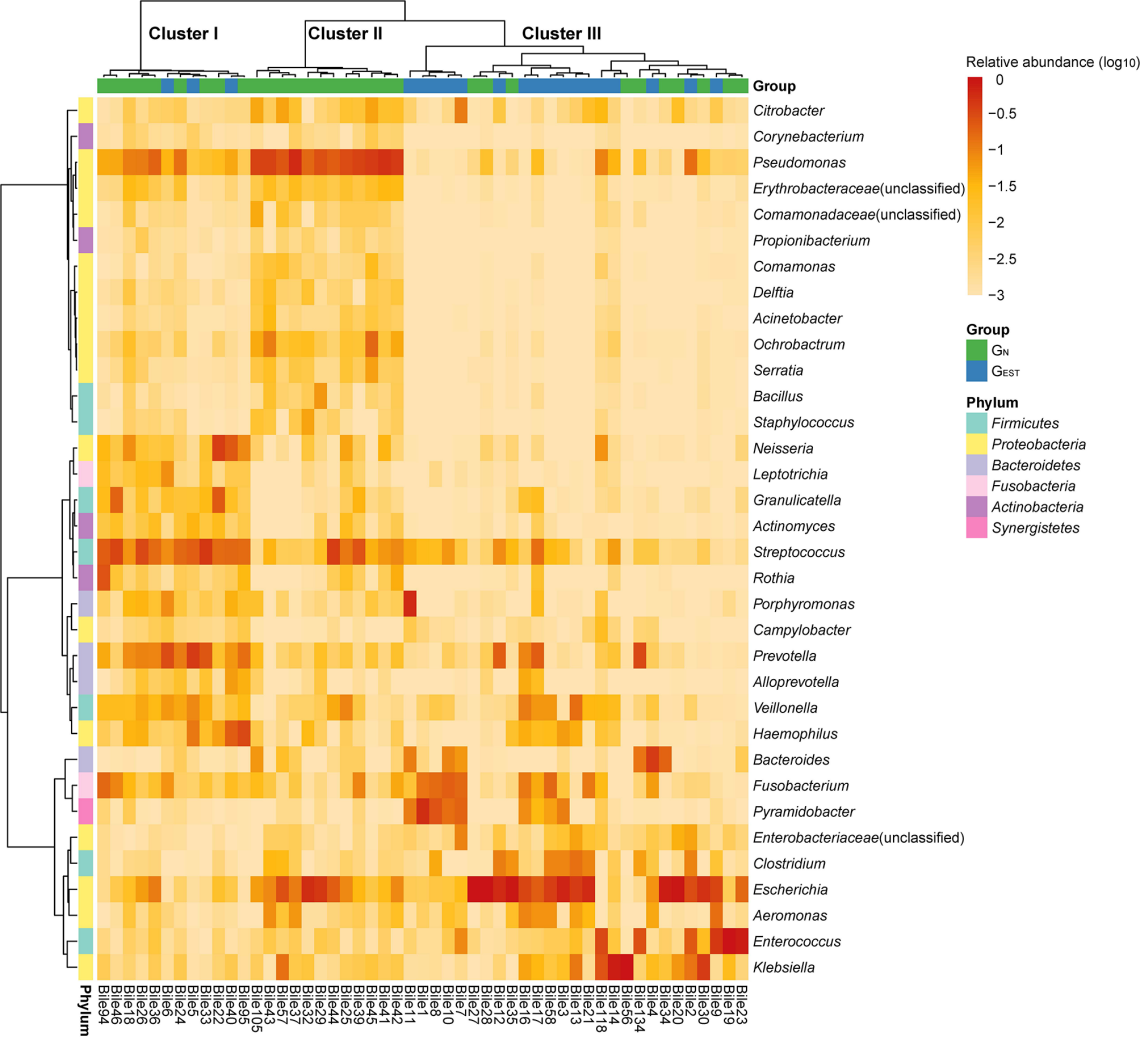
Distribution of highly prevalent genera in the G_EST_ and G_N_ groups. Only genera detected in at least 20% samples with a relative abundance ≥0.1% were displayed. The heatmap color scale quantifies the log_10_ relative abundance of each genus.

For the patients without AT before ERCP, we denoted the 20 patients with an EST history as the patient group G_EST_ and the 31 patients with an intact sphincter of Oddi as G_N_. In G_EST_ and G_N_, the identified biliary bacteria were mainly from six phyla, *Firmicutes*, *Proteobacteria*, *Bacteroidetes*, *Fusobacteria*, *Actinobacteria*, and *Synergistetes* (**Figure 1**). *Proteobacteria* was the most prevalent and had a mean RA of 53.12% (SD 30.24%), followed by *Firmicutes* (23.65% ± 21.02%). At the genus level, a total of 213 genera were observed with an RA ≥ 0.1%. Thirty-four prevalent genera existed in at least 10 (20%) samples (**Figure 1**, **Table S2**), and four of them, *Streptococcus*, *Escherichia*, *Fusobacterium*, and *Pseudomonas*, had a prevalence ≥70%. The prevalent bacterial genera were also identified in previous studies (Shen et al., 2015; Ye et al., 2016).

The results also show substantial heterogeneity in the bacterial distribution among non-treated patients, which was consistent with our previous findings (Shen et al., 2015). In the genera with an RA > 1% in at least one patient, we observed 29 genera in less than five patients (**Table S3**), 19 of which were patient-specific. Notably, among these genera, *Edwardsiella*, *Akkermansia*, *Lactobacillus*, *Morganella*, *Helicobacter*, and *Clostridium XlVa* had an RA higher than 10% in specific patients. Moreover, to the best of our knowledge, three of the rare genera, *Tepidimonas*, *Aquabacterium*, and *Megamonas*, were first discovered in the biliary samples of choledocholithiasis patients. The presence of the newly identified genera was reliable, as all had an RA ≥ 1.16% in at least one patient.

The results of bacterial cultivation were consistent with the results of NGS, but bacterial cultivation was much less sensitive than NGS in identifying bacteria. A total of 11 different bacterial species were cultured from 32 samples in the four patient groups (**Table S4**), and these samples all had corresponding OTUs at the genus level. In these samples validated by cultivation, 62.16% of the corresponding OTUs had an RA ≥ 10%, and 81.08% had an RA ≥ 1%. The majority of the cultured species belonged to the prevalent genera *Escherichia*, *Klebsiella*, *Enterococcus*, *Citrobacter*, *Proteus*, and *Pseudomonas*. Notably, species of two rare genera, *Morganella morganii* and *Edwardsiella tarda*, were also successfully cultivated.

### Clusters of Biliary Microbial Compositions and Their Association With Endoscopic Sphincterotomy

Based on the biliary bacterial compositions, we performed an unsupervised clustering analysis of these samples (**Figure 1**). We identified three clusters that were characterized by the bacteria normally colonized in the oral cavity/respiratory tract (cluster I), environment/skin (cluster II), and gastrointestinal tract (cluster III) (**Table S5**). Cluster I and cluster III were consistent with our previous pilot study of the biliary microbiota (Shen et al., 2015), which failed to identify cluster II due to a limited sample size (*n* = 15). The abundant and enriched genera in each cluster were investigated. In cluster I, the top five abundant and significantly enriched bacterial genera (two-side Wilcoxon rank-sum test, FDR < 0.05) were *Streptococcus* (20.73 ± 11.44%), *Prevotella* (13.45 ± 12.44%), *Neisseria* (7.21 ± 11.62%), *Haemophilus* (6.83 ± 10.15%), and *Granulicatella* (4.62 ± 7.41%). Cluster II was highly abundant in *Pseudomonas* (35.37 ± 12.40%), followed by *Ochrobactrum* (3.86 ± 4.99%) and *Serratia* (1.09 ± 1.38%). These genera were significantly enriched (FDR < 0.05) in cluster II and have been reported to colonize natural water or soil. *Propionibacterium*, which is often isolated from human skin, was also enriched in cluster II (0.21 ± 0.15%; FDR < 0.05).

Compared with the most abundant genera in clusters I and II, the most abundant genera in cluster III were typical gastrointestinal inhabitants, such as *Escherichia* (33.78 ± 34.15%), *Enterococcus* (11.82 ± 24.74%), *Klebsiella* (10.80 ± 24.30%), *Fusobacterium* (4.56 ± 6.73%), and *Bacteroides* (3.70 ± 8.89%). However, although these genera had a high RA, they were not significantly enriched. In cluster III, *Porphyromonas* (2.34 ± 11.36%) and an unclassified *Enterobacteriaceae* (1.25 ± 2.22%) were significantly enriched (FDR < 0.05).

Intriguingly, samples from G_N_ dominated cluster I (9 of 12) and cluster II (12 of 12), while cluster III was mainly enriched with samples from G_EST_ (17 of 27). Thus, the distribution of G_N_ and G_EST_ were significantly biased among the three clusters (two-side Fisher’s exact test of a 2 × 3 contingency table, *P* = 0.00019). We combined clusters I and II and compared the pooled clusters with cluster III, which indicated that the association with EST history was also significant (two-side Chi-square test of a 2 × 2 contingency table, *P* = 0.00042). These results indicate that the history of EST was associated with the cluster III microbiota, which was characterized by abundant gastrointestinal bacteria.

### Comparison Between Biliary Microbiota With and Without Endoscopic Sphincterotomy History

**Figure 2:**
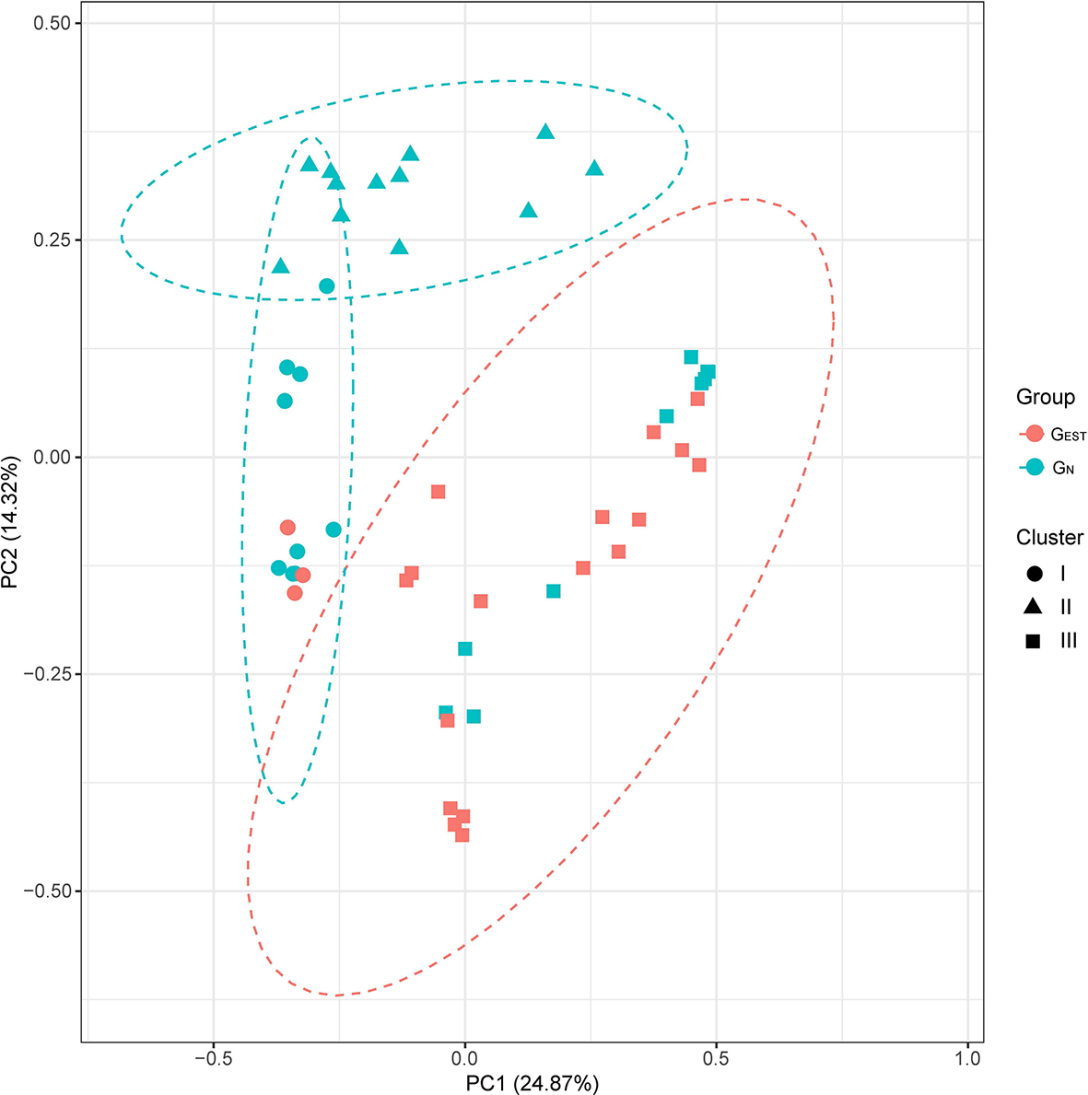
Principal coordinate analysis of the G_EST_ and G_N_ groups. The β-diversities were compared based on Bray-Curtis dissimilarity. The results of unsupervised clustering analysis are also presented.

A comparative study between G_EST_ and G_N_ showed that biliary samples from the two groups had a comparable α-diversity but a significantly different β-diversity, namely, the biliary microbiota of patients with a history of EST did not have an elevated taxonomic diversity but was more likely to undergo a shift in bacterial composition than the biliary microbiota of patients without a history of EST. We measured the α-diversity of G_N_ and G_EST_ by using species richness, phylogenetic diversity, abundance, and evenness with three indices (Chao1, PD_whole_tree, and Shannon index). We found no statistically significant differences (Wilcoxon rank-sum test, *P* > 0.05, **Figure S2**). In contrast, analysis of similarities (ANOSIM) revealed that G_N_ and G_EST_ differed significantly in microbiota composition by using β-diversity indices of Bray-Curtis dissimilarity (*P* = 0.001, **Figure 2**), unweighted UniFrac distance (*P* = 0.001) and weighted UniFrac distance (*P* = 0.03). Adonis and PERMANOVA analyses exhibited similar results (**Table S6**). A principal coordinates analysis (PCoA) based on Bray-Curtis dissimilarity indicated that the first and second principal components (PC1 and PC2) contributed 24.87 and 14.32% of the total variance between G_N_ and G_EST_ (**Figure 2**).

**Figure 3:**
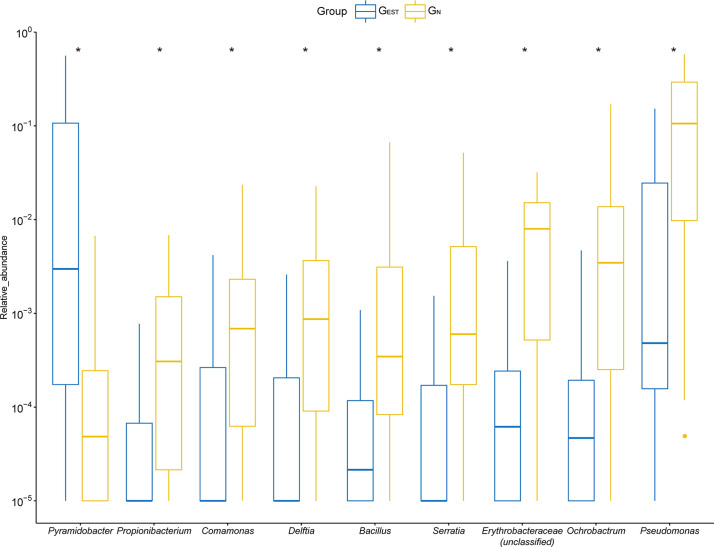
The differential genera detected between samples from the G_EST_ and G_N_ groups. For optimal visualization, a transformation of log10 (relative abundance + 0.00001) was employed. Boxes represent the interquartile range (IQR) between the first and third quartiles (25th and 75th percentiles, respectively). Lines inside denote the median, and whiskers denote the most extreme values within 1.5 times IQR from the first and third quartiles. Outlier values are represented as points. *FDR < 0.05.

Among the prevalent genera that existed in ≥20% patients, we found nine genera that had significantly different RAs between G_N_ and G_EST_ (Wilcoxon test, FDR < 0.05, **Figure 3**, **Table S2**). One genus, *Pyramidobacter*, was significantly enriched in G_EST_ (7.72 ± 13.64%, FDR = 0.036), whereas the remaining eight genera had a reduced RA in G_EST_. *Pyramidobacter* was detected in 11 samples (55%) from G_EST_, mostly with a high RA (9 samples >1%). In contrast, in 24 of 31 G_N_ samples, we failed to identify *Pyramidobacter*, and the rest had a low RA (0.1–1%). This result indicates that abundant *Pyramidobacter* in biliary samples was specific for patients with an EST history. Biliary *Pyramidobacter* was first discovered in a gallstone patient with an EST history (Shen et al., 2015). Representative species from *Pyramidobacter* were reported to be isolated from the oral cavity and small intestine (Downes et al., 2009; Marchandin et al., 2010; Rôças and Siqueira, 2010). We previously showed the coexistence of *Pyramidobacter* bile and gastric and duodenal fluid in six patients with an EST history, suggesting the possibility of retrograde infection of *Pyramidobacter* (Ye et al., 2016). Among the bacteria that exhibited a decline in RA in G_EST_ (**Figure 3**), consistent with the clustering analysis, six out of eight originated from the environment or human skin.

We further compared the predicted profiles of KEGG pathways and KEGG Orthology (KO) terms of biliary microbial communities. A total of 61 pathways were differentially expressed between the G_N_ and G_EST_ samples (**Table S7**). Notably, the glutathione metabolic pathway, which could be employed by some bacteria to address the induced oxidative stresses by bile acids, was depleted in recurrent patients, Among the 51 differential KO terms (**Table S8**), there were 23 ones associated with bacterial metabolism. Glycogen phosphorylase (K00688) with increased representation in recurrent patients was involved in biofilm formation of *E. coli*, indicating a potential role in gallstone formation.

### The Biliary Microbiota After Antibiotic Treatment

Some patients received AT (denoted as AT patients) 1–5 days before ERCP due to the presence of acute suppurative cholangitis. We focused on the nine significantly distributed bacterial genera identified in G_N_ and G_EST_. We found that for these AT patients, seven of the genera maintained an association with EST history but with a reduced significance (**Table S2**). For instance, *Pyramidobacter* was still the most significantly enriched genera among AT patients with a history of EST compared with those patients without (Wilcoxon rank-sum test, *P* = 0.00011, FDR = 0.062). Two genera, *Fusobacterium* and *Citrobacter*, had a markedly reduced RA in AT patients (both *P* < 0.05) such that the association with a history of EST was not significant or even reversed. After AT, the ANOSIM based on Bray-Curtis dissimilarity (β-diversity) could not separate the samples with or without EST history (*P* = 0.409). In summary, for patients treated with antibiotics before bile collection, the EST-associated pattern disappeared for overall biliary microbiota but was preserved for specific bacterial genera.

## Discussion

By employing 16S rRNA sequencing technology, we found that the biliary microbial community of primary choledocholithiasis patients was associated with a history of an EST operation. We grouped the discovered biliary microbiota into three clusters, and the biliary microbiota of patients with a history of EST were significantly enriched in the cluster characterized by gastrointestinal bacteria. Although most genera that differed in samples from patients with a history of EST showed a reduced abundance, *Pyramidobacter* was the only significantly increased genus (FDR < 0.05) in the EST group and exhibited high specificity. In this study, we excluded patients with secondary choledocholithiasis, thus the impact of EST on the biliary microbiota of this sort of patients needs further investigation.

EST could be a vital factor that determines the shift in biliary bacteria. It could cause damage to the sphincter of Oddi and thus alter the microenvironment of the bile duct. Sphincter of Oddi damage after EST would lead to DBR. With the regurgitation of the duodenal contents, gut bacteria may enter the biliary duct and affect the ecological dynamics to a prolithogenic state, which may ultimately lead to gallstone recurrence. In a prospective case-control study, Zhang et al. found that DBR rates in patients with no, single, or multiple CBDS recurrences after EST were 15.6, 60.9, and 88.9%, respectively (Zhang et al., 2015). Therefore, DBR is correlated with CBDS recurrence in patients who had previously undergone ERCP. In our study, the frequency of duodenal-biliary reflux hasn’t been evaluated, which will be investigated in the further study.

Higher abundances of *Pyramidobacter* were observed in recurrent patients than in patients without EST history in our study. The archetype species *P. piscolens* encodes phospholipase proteins (GenBank: WP_009165038.1 and EFB90440.1), which might be involved in gallstone formation. With regard to bile resistance, the genome contains genes related to riboflavin metabolism, i.e., riboflavin synthase (WP_009165801.1), the riboflavin biosynthesis protein RibD (K11752, WP_050768911.1, and EFB89795.1), and the riboflavin biosynthesis protein RibF (K11753, WP_083798262.1, and EFB90920.1).

EST could also affect the functions of biliary bacteria in response to environmental modifications. Some gut bacteria might enter the biliary tract after EST operation, which would cause retrograde infection of the bile duct. These bacteria could use the glutathione metabolic pathway, riboflavin metabolic pathway, and cysteine/methionine metabolic pathway to address the induced oxidative stresses. The glutathione metabolic pathway was depleted, while two KEGG Orthology (KO) terms related to riboflavin metabolism were elevated in recurrent patients, suggesting the compensation of oxidative stress responses of the biliary bacteria in the absence of one particular pathway. There were 23 metabolism-related KO terms. Eleven genes were involved in the biosynthesis of secondary metabolites (ko01110), and eight KO terms were related to the biosynthesis of antibiotics (ko01130). Five members from microbial metabolism in diverse environments (ko01120) also had significant differences between the two groups, which suggested that the alteration in the biliary microenvironment might impact the bacterial responses to stresses.

Since the introduction of ERCP and EST nearly 50 years ago, in addition to their therapeutic effects, clinicians have performed many studies on their short-term and long-term complications, especially on the pathogenesis of long-term complications, such as cholangiocarcinoma and recurrent common bile duct stones. Many studies have observed that bacteria might play a key role in the development of these complications, most of which are due to the destruction of the function of sphincter of Oddi by EST. Nonetheless, little is known about the alterations in the bacterial flora in the human bile duct after the function of the papillary sphincter is damaged, and the potential underlying pathogenic role of bacteria in the bile duct remains largely unknown. This study has observed a shift in the biliary microbiota of gallstone patients induced by EST, which could provide evidence for the undesirable consequences due to this safe, effective, and yet invasive clinical procedure. This study also highlights the importance of protecting the normal functions of the sphincter of Oddi in future treatments.

## Data Availability Statement

The datasets presented in this study can be found in online repositories. The names of the repository/repositories and accession number(s) can be found at: https://www.ncbi.nlm.nih.gov/, PRJNA543184.

## Ethics Statement

The studies involving human participants were reviewed and approved by the Institutional Review Board of Affiliated Hangzhou First People’s Hospital, Zhejiang University School of Medicine. The patients/participants provided their written informed consent to participate in this study.

## Author Contributions

MN and XZ conceived and designed the study. LF, JY, and QL collected the samples and patient information. JZ, DX, and HJ conducted the experiments. HS, FY, and MN generated the sequencing data. FY, MN, and HS analyzed and interpreted the data. FY and MN wrote the manuscript with contributions from all other authors. All authors contributed to the article and approved the submitted version.

## Funding

This work was supported by the Natural Science Foundation of Zhejiang Province (Grant No. LY17H030003), the National Natural Science Foundation of China (Grant Nos. 31701158, 31870079, and 81602325), the Zhejiang Medical and Health Science and Technology Plan (Grant Nos. WKJ-ZJ-2136 and 2019RC068), and the Hangzhou Medical and Health Science and Technology Plan (Grant Nos. 2016ZD01, OO20190610, and A20200174). MN was supported by the Beijing Nova Program (Grant No. Z181100006218114). The funders had no role in the study design, data collection and interpretation, or the decision to submit the work for publication.

## Conflict of Interest

The authors declare that the research was conducted in the absence of any commercial or financial relationships that could be construed as a potential conflict of interest.

## Supplementary Material

Supplementary Figure 1 Rarefaction curves measured by the Chao1 **(A)** and Shannon **(B)** indices.

Supplementary Figure 2 The comparison of α-diversities of GN and GEST samples. Chao1, PD_whole_tree and Shannon indices were employed.
