# Supplementary figures and images for: Biliary Microbial Structure of Gallstone Patients With a History of Endoscopic Sphincterotomy Surgery
Source: Front Cell Infect Microbiol. 2021 Jan 27;10:594778. doi: 10.3389/fcimb.2020.594778 (PMC7873689; doi:10.3389/fcimb.2020.594778)

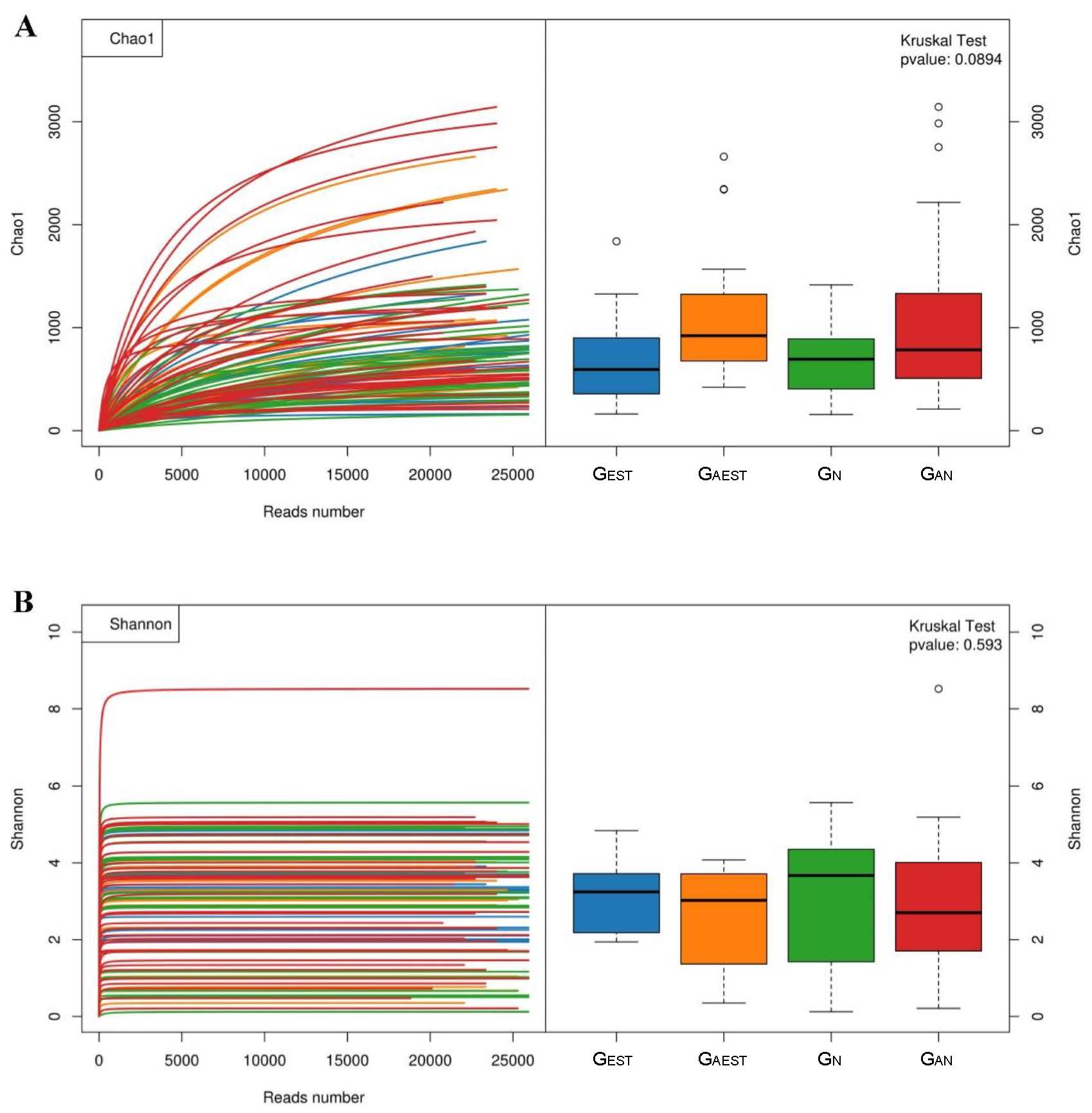

Supplement: Supplementary Figure 1 — Rarefaction curves measured by the Chao1 (A) and Shannon (B) indices. [file Image_1.jpeg]

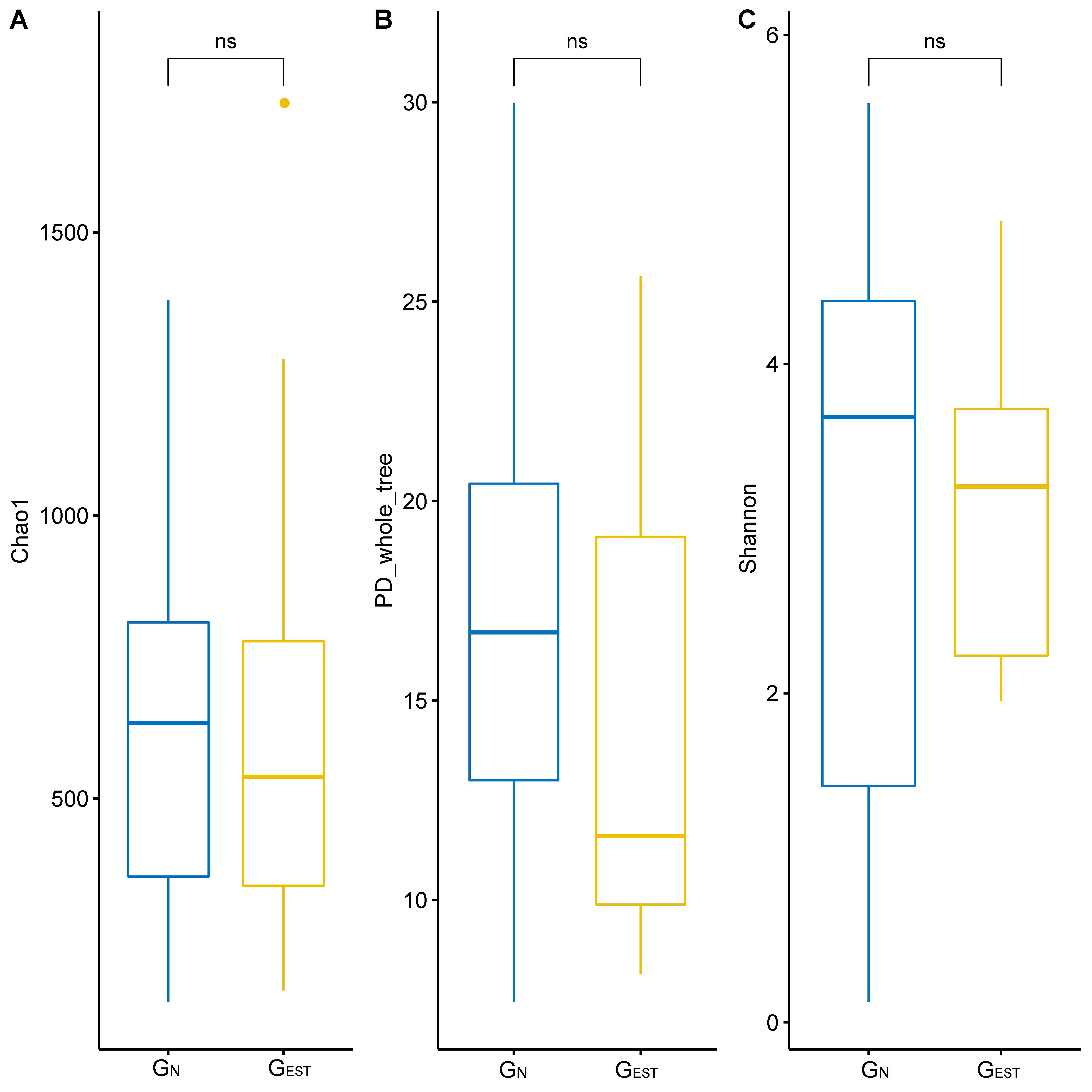

Supplement: Supplementary Figure 2 — The comparison of α-diversities of GN and GEST samples. Chao1, PD_whole_tree and Shannon indices were employed. [file Image_2.jpeg]
