# Supplementary material for: Biliary Microbial Structure of Gallstone Patients With a History of Endoscopic Sphincterotomy Surgery
Source: Front Cell Infect Microbiol. 2021 Jan 27;10:594778. doi: 10.3389/fcimb.2020.594778 (PMC7873689; doi:10.3389/fcimb.2020.594778)
Supplement: Supplementary file 7 [file Table_5.docx]

**Table S5.** The reported colonization sites of relevant bacteria.

| **Genus** | **Colonization site** | **Reference** |
| --- | --- | --- |
| *Streptococcus* | human oral cavity and nasopharynx/gut | ([Nobbs et al., 2015](#_ENREF_30)) ([Kaci et al., 2014](#_ENREF_19); [Keravec et al., 2015](#_ENREF_21); [Mason et al., 2018](#_ENREF_25)) |
| *Escherichia* | gut | ([Mitchell and Hill, 2000](#_ENREF_26)) |
| *Pseudomonas* | respiratory tract/urinary tract/ environmental | ([Cohen et al., 2017](#_ENREF_10); [von Klitzing et al., 2017](#_ENREF_44); [Suarez-Cuartin et al., 2018](#_ENREF_41)) |
| *Enterococcus* | gut | ([Ford et al., 2017](#_ENREF_16)) |
| *Klebsiella* | oral cavity/skin/gut/ environmental | ([Mättö et al., 1996](#_ENREF_24); [Davey and O'Toole G, 2000](#_ENREF_12)) |
| *Citrobacter* | gut/ respiratory tract/ urinary tract | ([Arens and Verbist, 1997](#_ENREF_3); [Begley et al., 2005](#_ENREF_5); [Liu et al., 2016](#_ENREF_23)) |
| *Prevotella* | oral cavity/gut/respiratory tract/ vagina | ([Kaufman et al., 1989](#_ENREF_20); [Cetta, 1991](#_ENREF_9); [Watnick et al., 2001](#_ENREF_45); [Stewart et al., 2006](#_ENREF_40); [Keravec et al., 2015](#_ENREF_21); [Beamer et al., 2017](#_ENREF_4)) |
| *Veillonella* | oral cavity/gut/ respiratory tract | ([Tronel et al., 2001](#_ENREF_42); [Watnick et al., 2001](#_ENREF_45); [Keravec et al., 2015](#_ENREF_21); [Mason et al., 2018](#_ENREF_25)) |
| *Fusobacterium* | oral cavity/gut/ respiratory tract | ([Prouty et al., 2002](#_ENREF_35); [Keravec et al., 2015](#_ENREF_21); [Mason et al., 2018](#_ENREF_25)) |
| *Ochrobactrum* | stomach/respiratory tract/environmental | ([de Dios Caballero et al., 2016](#_ENREF_13); [Kulkarni et al., 2017](#_ENREF_22))  https://www.sciencedirect.com/topics/medicine-and-dentistry/ochrobactrum-anthropi |
| *Granulicatella* | oral cavity/respiratory tract/ intestine/genitourinary tract | ([Siqueira and Rôças, 2006](#_ENREF_39); [Vandana et al., 2010](#_ENREF_43); [Farrell et al., 2011](#_ENREF_15); [Keravec et al., 2015](#_ENREF_21); [Mason et al., 2018](#_ENREF_25)) |
| *Clostridium* | gut | ([Shimizu et al., 2002](#_ENREF_38); [Crobach et al., 2018](#_ENREF_11)) |
| *Porphyromonas* | oral cavity/ respiratory tract/ vaginal and intestinal tracts | ([Keravec et al., 2015](#_ENREF_21); [Mason et al., 2018](#_ENREF_25)) |
| Campylobacter | oral cavity/ respiratory tract/environmental/gut | ([Keravec et al., 2015](#_ENREF_21); [Amer et al., 2017](#_ENREF_2); [Bloomfield et al., 2017](#_ENREF_6); [Johnson et al., 2017](#_ENREF_18); [Mason et al., 2018](#_ENREF_25)) |
| Actinomyces | oral cavity/respiratory tract/ gastrointestinal | ([Keravec et al., 2015](#_ENREF_21); [Mason et al., 2018](#_ENREF_25)) |
| Acinetobacter | skin/environmental | ([Seifert et al., 1993](#_ENREF_37); [Cohen et al., 2017](#_ENREF_10)) |
| Serratia | eye/gut/environmental/respiratory trac | ([Montagnani et al., 2015](#_ENREF_27); [Morillo et al., 2016](#_ENREF_28)) |
| Pyramidobacter | oral cavity | ([Downes et al., 2009](#_ENREF_14)) |
| Aeromonas | environmental | ([Nolla-Salas et al., 2017](#_ENREF_31)) |
| Alloprevotella | oral cavity | ([O'Toole and Kolter, 1998](#_ENREF_32); [Watnick et al., 2001](#_ENREF_45); [Nadkarni et al., 2015](#_ENREF_29)) |
| Comamonas | environmental | ([Parolin et al., 2016](#_ENREF_33); [Almuzara et al., 2017](#_ENREF_1); [Cohen et al., 2017](#_ENREF_10)) |
| Haemophilus | oral cavity/ respiratory tract | ([Keravec et al., 2015](#_ENREF_21); [Mason et al., 2018](#_ENREF_25)) |
| Delftia | environmental | ([Ranc et al., 2018](#_ENREF_36)) |
| Corynebacterium | oral cavity/ respiratory tract/skin/ mucosa/gut | ([Keravec et al., 2015](#_ENREF_21); [Bomar et al., 2016](#_ENREF_7); [Mason et al., 2018](#_ENREF_25)) |
| Rothia | oral cavity/ respiratory tract | ([Keravec et al., 2015](#_ENREF_21); [Amer et al., 2017](#_ENREF_2); [Mason et al., 2018](#_ENREF_25)) |
| Bacteroides | human intestinal tract | ([Gregory et al., 2015](#_ENREF_17)) |
| Neisseria | respiratory tract/ human oral cavity and nasopharynx | ([Keravec et al., 2015](#_ENREF_21)) |
| Leptotrichia | respiratory tract/oral cavity | ([Keravec et al., 2015](#_ENREF_21); [Amer et al., 2017](#_ENREF_2)) |
| Bacillus | gut | ([Piewngam et al., 2018](#_ENREF_34)) |
| Staphylococcus | skin/mucosa/environmental | ([Byrd et al., 2018](#_ENREF_8)) |
| Propionibacterium | skin | ([Byrd et al., 2018](#_ENREF_8)) |

**References**

Almuzara, M., Barberis, C., Veiga, F., Bakai, R., Cittadini, R., Vera Ocampo, C., et al. (2017). Unusual presentations of Comamonas kerstersii infection. *New Microbes New Infect* 19**,** 91-95. doi: 10.1016/j.nmni.2017.07.003

Amer, A., Galvin, S., Healy, C.M., and Moran, G.P. (2017). The Microbiome of Potentially Malignant Oral Leukoplakia Exhibits Enrichment for Fusobacterium, Leptotrichia, Campylobacter, and Rothia Species. *Front Microbiol* 8**,** 2391. doi: 10.3389/fmicb.2017.02391.

Arens, S., and Verbist, L. (1997). Differentiation and susceptibility of Citrobacter isolates from patients in a university hospital. *Clin Microbiol Infect* 3(1)**,** 53-57. doi: S1198-743X(15)60158-6 [pii].

Beamer, M.A., Austin, M.N., Avolia, H.A., Meyn, L.A., Bunge, K.E., and Hillier, S.L. (2017). Bacterial species colonizing the vagina of healthy women are not associated with race. *Anaerobe* 45**,** 40-43. doi: S1075-9964(17)30041-0 [pii]

Begley, M., Gahan, C.G., and Hill, C. (2005). The interaction between bacteria and bile. *Fems Microbiology Reviews* 29(4)**,** 625-651.

Bloomfield, S.J., Midwinter, A.C., Biggs, P.J., French, N.P., Marshall, J.C., Hayman, D.T.S., et al. (2017). Long-term Colonization by Campylobacter jejuni Within a Human Host: Evolution, Antimicrobial Resistance, and Adaptation. *J Infect Dis* 217(1)**,** 103-111. doi: 10.1093/infdis/jix561

Bomar, L., Brugger, S.D., Yost, B.H., Davies, S.S., and Lemon, K.P. (2016). Corynebacterium accolens Releases Antipneumococcal Free Fatty Acids from Human Nostril and Skin Surface Triacylglycerols. *MBio* 7(1)**,** e01725-01715. doi: 10.1128/mBio.01725-15

Byrd, A.L., Belkaid, Y., and Segre, J.A. (2018). The human skin microbiome. *Nat Rev Microbiol* 16(3)**,** 143-155. doi: 10.1038/nrmicro.2017.157

Cetta, F. (1991). The role of bacteria in pigment gallstone disease. *Annals of surgery* 213(4)**,** 315.

Cohen, R., Babushkin, F., Shimoni, Z., Cohen, S., Litig, E., Shapiro, M., et al. (2017). Water faucets as a source of Pseudomonas aeruginosa infection and colonization in neonatal and adult intensive care unit patients. *Am J Infect Control* 45(2)**,** 206-209. doi: S0196-6553(16)30683-6 [pii]

Crobach, M.J.T., Vernon, J.J., Loo, V.G., Kong, L.Y., Pechine, S., Wilcox, M.H., et al. (2018). Understanding Clostridium difficile Colonization. *Clin Microbiol Rev* 31(2). doi: 10.1128/CMR.00021-17

Davey, M.E., and O'Toole G, A. (2000). Microbial biofilms: from ecology to molecular genetics. *Microbiol Mol Biol Rev* 64(4)**,** 847-867.

de Dios Caballero, J., Del Campo, R., Royuela, A., Sole, A., Maiz, L., Olveira, C., et al. (2016). Bronchopulmonary infection-colonization patterns in Spanish cystic fibrosis patients: Results from a national multicenter study. *J Cyst Fibros* 15(3)**,** 357-365. doi: 10.1016/j.jcf.2015.09.004

Downes, J., Vartoukian, S.R., Dewhirst, F.E., Izard, J., Chen, T., Yu, W.-H., et al. (2009). Pyramidobacter piscolens gen. nov., sp. nov., a member of the phylum ‘Synergistetes’ isolated from the human oral cavity. *International journal of systematic and evolutionary microbiology* 59(5)**,** 972-980.

Farrell, J.J., Zhang, L., Zhou, H., Chia, D., Elashoff, D., Akin, D., et al. (2011). Variations of oral microbiota are associated with pancreatic diseases including pancreatic cancer. *Gut***,** gutjnl-2011-300784.

Ford, C.D., Gazdik, M.A., Lopansri, B.K., Webb, B., Mitchell, B., Coombs, J., et al. (2017). Vancomycin-Resistant Enterococcus Colonization and Bacteremia and Hematopoietic Stem Cell Transplantation Outcomes. *Biol Blood Marrow Transplant* 23(2)**,** 340-346. doi: S1083-8791(16)30517-1 [pii]

Gregory, K.E., LaPlante, R.D., Shan, G., Kumar, D.V., and Gregas, M. (2015). Mode of Birth Influences Preterm Infant Intestinal Colonization With Bacteroides Over the Early Neonatal Period. *Adv Neonatal Care* 15(6)**,** 386-393. doi: 10.1097/ANC.0000000000000237.

Johnson, T.J., Shank, J.M., and Johnson, J.G. (2017). Current and Potential Treatments for Reducing Campylobacter Colonization in Animal Hosts and Disease in Humans. *Front Microbiol* 8**,** 487. doi: 10.3389/fmicb.2017.00487.

Kaci, G., Goudercourt, D., Dennin, V., Pot, B., Doré, J., Ehrlich, S.D., et al. (2014). Anti-inflammatory properties of Streptococcus salivarius, a commensal bacterium of the oral cavity and digestive tract. *Applied and Environmental Microbiology* 80(3)**,** 928-934.

Kaufman, H.S., Magnuson, T.H., Lillemoe, K.D., Frasca, P., and Pitt, H.A. (1989). The role of bacteria in gallbladder and common duct stone formation. *Annals of surgery* 209(5)**,** 584.

Keravec, M., Mounier, J., Prestat, E., Vallet, S., Jansson, J.K., Burgaud, G., et al. (2015). Insights into the respiratory tract microbiota of patients with cystic fibrosis during early Pseudomonas aeruginosa colonization. *Springerplus* 4**,** 405. doi: 10.1186/s40064-015-1207-0

Kulkarni, G., Gohil, K., Misra, V., Kakrani, A.L., Misra, S.P., Patole, M., et al. (2017). Multilocus sequence typing of Ochrobactrum spp. isolated from gastric niche. *J Infect Public Health* 10(2)**,** 201-210. doi: S1876-0341(16)30031-4 [pii]

Liu, Z., Man, S.M., Zhu, Q., Vogel, P., Frase, S., Fukui, Y., et al. (2016). DOCK2 confers immunity and intestinal colonization resistance to Citrobacter rodentium infection. *Sci Rep* 6**,** 27814. doi: 10.1038/srep27814

Mättö, J., Saarela, M., Troil‐Lindén, B.v., Könönen, E., Jousimies‐Somer, H., Torkko, H., et al. (1996). Distribution and genetic analysis of oral Prevotella intermedia and Prevotella nigrescens. *Oral microbiology and immunology* 11(2)**,** 96-102.

Mason, M.R., Chambers, S., Dabdoub, S.M., Thikkurissy, S., and Kumar, P.S. (2018). Characterizing oral microbial communities across dentition states and colonization niches. *Microbiome* 6(1)**,** 67. doi: 10.1186/s40168-018-0443-2

Mitchell, J.L., and Hill, S.L. (2000). Immune response to Haemophilus parainfluenzae in patients with chronic obstructive lung disease. *Clinical and diagnostic laboratory immunology* 7(1)**,** 25-30.

Montagnani, C., Cocchi, P., Lega, L., Campana, S., Biermann, K.P., Braggion, C., et al. (2015). Serratia marcescens outbreak in a neonatal intensive care unit: crucial role of implementing hand hygiene among external consultants. *BMC Infect Dis* 15**,** 11. doi: 10.1186/s12879-014-0734-6

Morillo, A., Gonzalez, V., Aguayo, J., Carreno, C., Torres, M.J., Jarana, D., et al. (2016). A six-month Serratia marcescens outbreak in a Neonatal Intensive Care Unit. *Enferm Infecc Microbiol Clin* 34(10)**,** 645-651. doi: S0213-005X(16)00033-1 [pii]

Nadkarni, M.A., Chhour, K.L., Browne, G.V., Byun, R., Nguyen, K.A., Chapple, C.C., et al. (2015). Age-dependent changes in Porphyromonas gingivalis and Prevotella species/phylotypes in healthy gingiva and inflamed/diseased sub-gingival sites. *Clin Oral Investig* 19(4)**,** 911-919. doi: 10.1007/s00784-014-1301-7.

Nobbs, A.H., Jenkinson, H.F., and Everett, D.B. (2015). Generic determinants of Streptococcus colonization and infection. *Infect Genet Evol* 33**,** 361-370. doi: 10.1016/j.meegid.2014.09.018

Nolla-Salas, J., Codina-Calero, J., Valles-Angulo, S., Sitges-Serra, A., Zapatero-Ferrandiz, A., Climent, M.C., et al. (2017). Clinical significance and outcome of Aeromonas spp. infections among 204 adult patients. *Eur J Clin Microbiol Infect Dis* 36(8)**,** 1393-1403. doi: 10.1007/s10096-017-2945-4

O'Toole, G.A., and Kolter, R. (1998). Flagellar and twitching motility are necessary for Pseudomonas aeruginosa biofilm development. *Molecular Microbiology* 30(2)**,** 295-304.

Parolin, M., Baraldi, M., Valentini, E., Murer, L., and Vidal, E. (2016). Comamonas testosteroni-associated peritonitis in a pediatric peritoneal dialysis patient. *World J Nephrol* 5(2)**,** 220-223. doi: 10.5527/wjn.v5.i2.220.

Piewngam, P., Zheng, Y., Nguyen, T.H., Dickey, S.W., Joo, H.S., Villaruz, A.E., et al. (2018). Pathogen elimination by probiotic Bacillus via signalling interference. *Nature* 562(7728)**,** 532-537. doi: 10.1038/s41586-018-0616-y

Prouty, A., Schwesinger, W., and Gunn, J. (2002). Biofilm Formation and Interaction with the Surfaces of Gallstones by Salmonella spp. *Infection and Immunity* 70(5)**,** 2640-2649.

Ranc, A., Dubourg, G., Fournier, P.E., Raoult, D., and Fenollar, F. (2018). Delftia tsuruhatensis, an Emergent Opportunistic Healthcare-Associated Pathogen. *Emerging Infectious Diseases* 24(3)**,** 594-596. doi: 10.3201/eid2403.160939.

Seifert, H., Strate, A., Schulze, A., and Pulverer, G. (1993). Vascular Catheter—Related Bloodstream Infection Due to Acinetobacter johnsonii (Formerly Acinetobacter calcoaceticus var. lwoffii): Report of 13 Cases. *Clinical infectious diseases* 17(4)**,** 632-636.

Shimizu, T., Ohtani, K., Hirakawa, H., Ohshima, K., Yamashita, A., Shiba, T., et al. (2002). Complete genome sequence of Clostridium perfringens, an anaerobic flesh-eater. *Proceedings of the National Academy of Sciences* 99(2)**,** 996-1001.

Siqueira, J.F., and Rôças, I.N. (2006). Catonella morbi and Granulicatella adiacens: new species in endodontic infections. *Oral Surgery, Oral Medicine, Oral Pathology, Oral Radiology, and Endodontology* 102(2)**,** 259-264.

Stewart, L., Grifiss, J.M., Jarvis, G.A., and Way, L.W. (2006). Biliary bacterial factors determine the path of gallstone formation. *The American Journal of Surgery* 192(5)**,** 598-603.

Suarez-Cuartin, G., Giner, J., Merino, J.L., Rodrigo-Troyano, A., Feliu, A., Perea, L., et al. (2018). Identification of Pseudomonas aeruginosa and airway bacterial colonization by an electronic nose in bronchiectasis. *Respir Med* 136**,** 111-117. doi: S0954-6111(18)30037-4 [pii]

Tronel, H., Chaudemanche, H., Pechier, N., Doutrelant, L., and Hoen, B. (2001). Endocarditis due to Neisseria mucosa after tongue piercing. *Clinical microbiology and infection* 7(5)**,** 275-276.

Vandana, K.E., Mukhopadhyay, C., Rau, N.R., Ajith, V., and Rajath, P. (2010). Native valve endocarditis and femoral embolism due to Granulicatella adiacens: a rare case report. *Braz J Infect Dis* 14(6)**,** 634-636. doi: S1413-86702010000600015 [pii]

von Klitzing, E., Ekmekciu, I., Bereswill, S., and Heimesaat, M.M. (2017). Intestinal and Systemic Immune Responses upon Multi-drug Resistant Pseudomonas aeruginosa Colonization of Mice Harboring a Human Gut Microbiota. *Front Microbiol* 8**,** 2590. doi: 10.3389/fmicb.2017.02590.

Watnick, P.I., Lauriano, C.M., Klose, K.E., Croal, L., and Kolter, R. (2001). The absence of a flagellum leads to altered colony morphology, biofilm development and virulence in Vibrio cholerae O139. *Molecular Microbiology* 39(2)**,** 223-235. doi: mmi2195 [pii].
